# Fifteen-year real-world pharmacovigilance of multiple sclerosis disease-modifying therapies: adverse event profiles, drug-specific safety signals and clinical implications

**DOI:** 10.3389/fneur.2026.1845881

**Published:** 2026-10-13

**Authors:** Sen Lin, Bei Zhang, Yingzhao Zhang, Xinyu Zhang, Yijia Li, Weiqing Zhang, Li He

**Affiliations:** 1 The Fourth Clinical Medical College, Guangzhou University of Chinese Medicine, Guangzhou, China; 2 The Chinese University of Hong Kong, Hong Kong, China; 3 Department of Oncology, Shenzhen Hospital (Futian) of Guangzhou University of Chinese Medicine, Shenzhen, China; 4 Department of Traditional Chinese Medicine, Shenzhen Longhua District People's Hospital, Shenzhen, China; 5 Department of Oncology, Shenzhen Traditional Chinese Medicine Hospital, Shenzhen, China

**Keywords:** adverse drug events, disease-modifying therapy, disproportionality analysis, FAERS, multiple sclerosis, pharmacovigilance

## Abstract

**Background:**

Disease-modifying therapies (DMTs) are the cornerstone of remission-phase management for multiple sclerosis (MS), a prevalent autoimmune neuroinflammatory disorder of the central nervous system. However, their immune-modulating mechanisms carry inherent risks of systemic and neurological adverse events (ADEs), which remain a major barrier to optimal long-term care. This study aimed to comprehensively characterize the real-world safety profiles of all major MS DMT classes and identify clinically actionable safety signals.

**Methods:**

We analyzed 15-year (2009Q1–2023Q4) ADE reports for 8 classes of FDA-approved MS DMTs from the FDA Adverse Event Reporting System (FAERS) database. Four disproportionality algorithms (ROR, PRR, BCPNN, MGPS) were used for signal detection. Time-to-onset (TTO) analysis with Weibull distribution and multivariate logistic regression were performed to assess temporal risk patterns and patient-specific risk factors for important medical events (IMEs).

**Results:**

A total of 498,847 ADE reports (1,048,575 events) were included, with nearly 50% involving IMEs. The most common ADEs were systemic symptoms, infections, liver injury and alopecia. Notably, progressive multifocal leukoencephalopathy (PML, ROR = 15.59) and spontaneous abortion (ROR = 2.54) were identified as high-risk severe events. Drug-specific analysis revealed distinct safety signatures: anti-CD20 antibodies carried the highest infection risk, natalizumab was strongly associated with PML (ROR = 45.62), and teriflunomide had the highest alopecia risk (ROR = 12.73). Approximately 70% of IMEs occurred within 2 years of treatment initiation. Multivariate analysis identified older age, male sex and longer treatment duration as independent risk factors for IMEs, while obesity was a protective factor.

**Conclusions:**

This long-term FAERS-based analysis identified potential safety signals associated with major MS DMTs, providing hypothesis-generating evidence to support clinical vigilance and prioritize further validation, but not to estimate incidence, comparative risk, or causality.

## 1 Introduction

Multiple sclerosis (MS) is a common autoimmune disease of the central nervous system, characterized by multifocal inflammatory demyelination in the brain and spinal cord (1). According to the 2020 Global Burden of Disease report, the global prevalence of MS has risen to 2.8 million people, marking a 30% increase since 2013. The male-to-female ratio of MS cases is approximately 1:2 globally, with some regions reporting a ratio as high as 1:4 (2). Although the exact pathogenesis of MS remains unclear, it is thought to be associated with genetic factors, smoking, obesity, diet, air pollution, and ultraviolet radiation exposure (3). Several hypotheses also suggest that latent human herpesviruses may play a key role in triggering autoimmune responses (4–6).

MS is classified into four major types: relapsing-remitting, primary progressive, secondary progressive, and progressive relapsing. Acute relapses are primarily managed with short courses of high-dose corticosteroids or plasma exchange therapy to control severe, disabling neurological damage. During the remission phase, disease-modifying therapies (DMTs) are the mainstay of treatment, with the goals of reducing relapse frequency, improving quality of life, and delaying disability progression through immune modulation (7). DMT drugs used in DMT include glatiramer acetate (GA), teriflunomide (TFD), Nuclear factor erythroid 2-related factor 2 (NRF2) agonists (diroximel fumarate, dimethyl fumarate), anti- integrin α4 monoclonal antibodies (natalizumab), anti-CD20 monoclonal antibodies (ocrelizumab, ofatumumab), anti-CD25 monoclonal antibodies (daclizumab), anti-CD52 monoclonal antibodies (alemtuzumab), Sphingosine 1-phosphate receptor (S1PR) modulators (fingolimod, siponimod, ozanimod) (6).

While these DMT drugs have demonstrated efficacy in reducing relapse rates and slowing disease progression, they are also associated with a range of potential adverse drug events (ADEs). Since DMT target multiple key components of the immune system, including B cells, T cells, natural killer cells, cytokines, and adhesion molecules, they often lead to ADEs that affect not only the immune system but also other organ systems. Common ADEs include an increased risk of infections, liver function abnormalities, systemic symptoms, and hematological changes (8). In addition to these routine ADEs, certain DMT drugs are associated with rare but severe ADEs.

For example, natalizumab (NTZ), an anti-integrin α4 monoclonal antibody, reduces neuroinflammation by blocking lymphocyte adhesion and subsequent transmigration across the blood-brain barrier (9). However, NTZ has been linked to an increased risk of progressive multifocal leukoencephalopathy (PML), a life-threatening demyelinating disease caused by JC polyomavirus infection of oligodendrocytes (10, 11). Due to this risk, the FDA has issued a black box warning for NTZ. Similarly, the anti-CD52 monoclonal antibody alemtuzumab has been reported to increase the risk of thyroid disorders and certain malignancies (8, 12), and is generally not considered a first-line treatment (13). Fingolimod, a first-generation S1PR modulator, carries a significant risk of bradycardia or atrioventricular block, with cardiovascular risks peaking within 6 to 20 h after the first dose, sometimes leading to sudden death (14, 15). Although siponimod, a second-generation S1PR modulator, has a lower cardiovascular risk than fingolimod, this concern remains significant (16). Thus, despite the substantial benefits of DMT in improving patient outcomes, safety concerns continue to be a critical focus in clinical practice.

Drug selection should be individualized, taking into account patient-specific factors, disease stage, and the safety and efficacy profiles of available treatments. However, the occurrence of ADEs can significantly affect treatment adherence and patients' quality of life. As DMTs become more widely used in the management of MS, the importance of systematic post-marketing safety surveillance has become increasingly recognized. ADEs not only affect treatment adherence but may also pose serious health risks, in some cases resulting in life-threatening outcomes. Pharmacovigilance systems serve as crucial tools for monitoring drug safety. By collecting and analyzing adverse-event reports, these systems can identify emerging safety concerns and generate hypotheses for further clinical and epidemiological investigation.

Accordingly, the primary objective of this study was to characterize the real-world adverse-event reporting profiles of major MS DMTs using more than 15 years of data from the FDA Adverse Event Reporting System (FAERS). Specifically, we aimed to: (1) describe the distribution of reported adverse events associated with individual DMTs; (2) identify drug–event combinations showing disproportionate reporting; (3) assess the consistency of detected signals across complementary disproportionality analyses; and (4) compare the safety-reporting patterns of DMTs with different mechanisms of action.

This study was designed as an exploratory, hypothesis-generating pharmacovigilance analysis. The primary purpose of disproportionality analysis is signal detection, that is, the identification of adverse events reported more frequently with a given drug than expected within the FAERS database. The observed signals should not be interpreted as estimates of clinical incidence or absolute risk, because FAERS does not provide a reliable denominator of exposed patients. In addition, disproportionality signals cannot establish a causal relationship between a DMT and an adverse event. Therefore, the clinical relevance of the identified signals was interpreted in conjunction with their statistical consistency, biological plausibility, and available evidence from clinical studies and the pharmacovigilance literature (17).

The findings are intended to provide a systematic overview of post-marketing safety signals associated with MS DMTs, support the prioritization of potential safety concerns for further investigation, and inform clinical vigilance and long-term treatment monitoring. They are not intended to replace comparative epidemiological studies, clinical trials, treatment guidelines, or individualized clinical decision-making.

## 2 Methods and materials

### 2.1 Data source and preprocessing

This study collected publicly accessible individual ADE reports from the FAERS database, spanning from the first quarter of 2009 to the fourth quarter of 2023. These reports were submitted by healthcare professionals, consumers, and pharmaceutical companies. The dataset consists of seven data tables: patient demographics and administrative information (DEMO), drug/biologic information (DRUG), adverse events (REAC), patient outcomes (OUTC), report sources (RPSR), drug therapy start and end dates (THER), and indications for use/diagnosis (INDI). The seven FAERS tables were linked using the case identifier and, where applicable, the report identifier and report version information. The DEMO, DRUG, REAC, OUTC, RPSR, THER, and INDI files were integrated according to the FAERS data structure. Variables were retained only when they could be reliably linked to an individual case report.

The DMT for MS primarily include eight drug categories: GA, TFD, NRF2 agonists, anti-α4 integrin monoclonal antibodies, anti-CD20 monoclonal antibodies, anti-CD25 monoclonal antibodies, anti-CD52 monoclonal antibodies, and S1PR modulators. The anti-CD20 monoclonal antibody category included ocrelizumab and ofatumumab. Although ublituximab is an anti-CD20 monoclonal antibody approved for the treatment of MS, it was excluded from the present comparative analysis because only 189 initial FAERS reports were identified, and the number of eligible reports after duplicate removal and data-quality processing was insufficient to support stable and interpretable signal estimation. Daclizumab was included in the anti-CD25 monoclonal antibody category because it had been previously authorized for the treatment of relapsing forms of MS in certain jurisdictions, including the European Union, during part of the study period. Although it was subsequently withdrawn worldwide because of serious safety concerns, FAERS includes historical post-marketing reports collected before its withdrawal. Accordingly, daclizumab-related signals in the present study reflect historical exposure and should not be interpreted as evidence of its current approval or routine clinical use in Europe or elsewhere.

We retrieved ADE reports for these selected MS DMTs that were approved in the United States during the study period or had relevant post-marketing reports in FAERS, focusing on reports involving one or more of these eight DMT. The specific search terms used for each drug can be found in Supplementary Table S1. Duplicate reports were removed following FAERS-recommended procedures. The specific drugs included in each category and the corresponding generic names used for data retrieval are provided in Supplementary Table S1.

ADEs were described using medical terms at both the system organ class (SOC) and preferred term (PT) levels, as defined by the Medical Dictionary for Regulatory Activities (version 27.0, https://www.meddra.org/). The MedDRA hierarchy was used to map each reported event to its corresponding PT and SOC. The PT level was used for specific event-level analyses, whereas the SOC level was used to summarize broader organ-system patterns. The MedDRA version and coding level were kept consistent across all analyses. In FAERS, ADEs are categorized into four groups: primary suspected (PS), second suspected (SS), concomitant (C), and interacting (I). To ensure the reliability of our analysis, only reports in which at least one selected DMT was classified as the primary suspect drug were included in the primary drug-specific analysis, while SS, C, and I reports were excluded from the study. The PS designation indicates the reporter's classification of the suspected role of the drug and does not establish a causal relationship between the drug and the reported event. This distinction was considered when interpreting all drug–event associations.

FAERS categorizes ADEs outcomes into seven levels: congenital anomaly (CA), death (DE), disability (DS), hospitalization (HO), life-threatening (LT), required intervention to prevent permanent impairment/damage (RI), and other (OT). Events classified as CA, DE, DS, HO, or LT are considered important medical events (IMEs), and this study focused on reports of IMEs. The RI and OT categories were not included in the primary IME definition because the present analysis focused on outcomes indicating serious medical consequences, consistent with the predefined outcome classification used in this study.

Reports from healthcare professionals, consumers, and pharmaceutical companies were retained in the primary analysis. Reporter type was summarized descriptively and considered a potential source of reporting heterogeneity. Reports with an unknown or unclassifiable reporter type were excluded from analyses involving reporter category but were excluded from the main signal analysis. Because FAERS is a spontaneous-reporting system, the number of reports should not be interpreted as the number of exposed patients. The analysis therefore used reporting counts and disproportionality measures rather than incidence rates.

Detailed data retrieval, collection, and screening processes are outlined in Figure 1. All statistical analyses and visualizations were performed using R software (v 4.3.2). The analysis code can be obtained from the corresponding author upon reasonable request.

**Figure 1:**
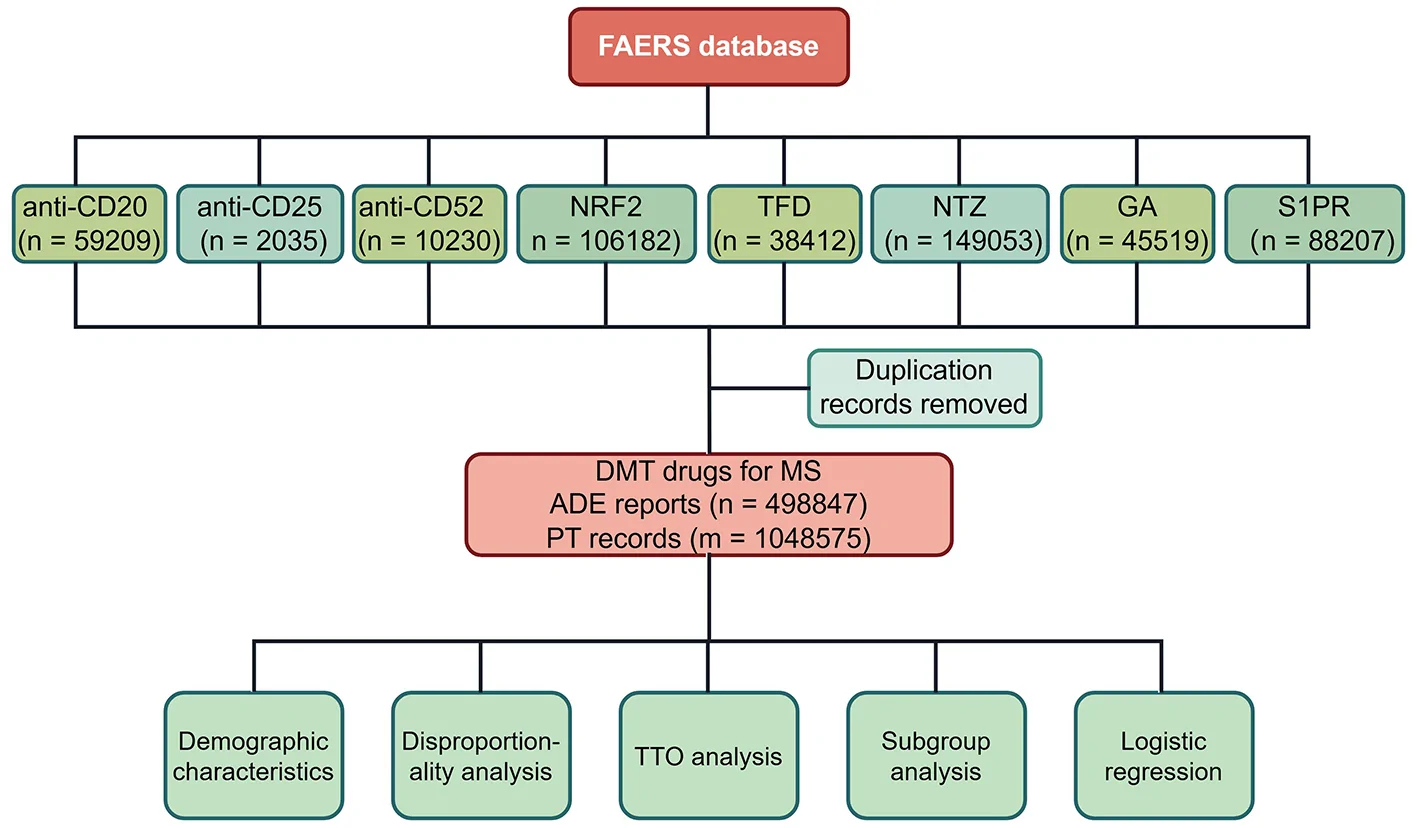
Detailed technical roadmap of this study.

### 2.2 Descriptive analysis

We first summarized the total annual number of adverse drug event reports from 2009 to 2023, as shown in Figure 2. After filtering ADE reports for the eight DMT drug categories, we conducted a descriptive analysis of the basic report information. This analysis included demographic information (sex, age, weight), reporter occupation (OCCP_COD), reporting country (REPORTER_COUNTRY), outcome (OUTC_COD), and year of report submission.

**Figure 2:**
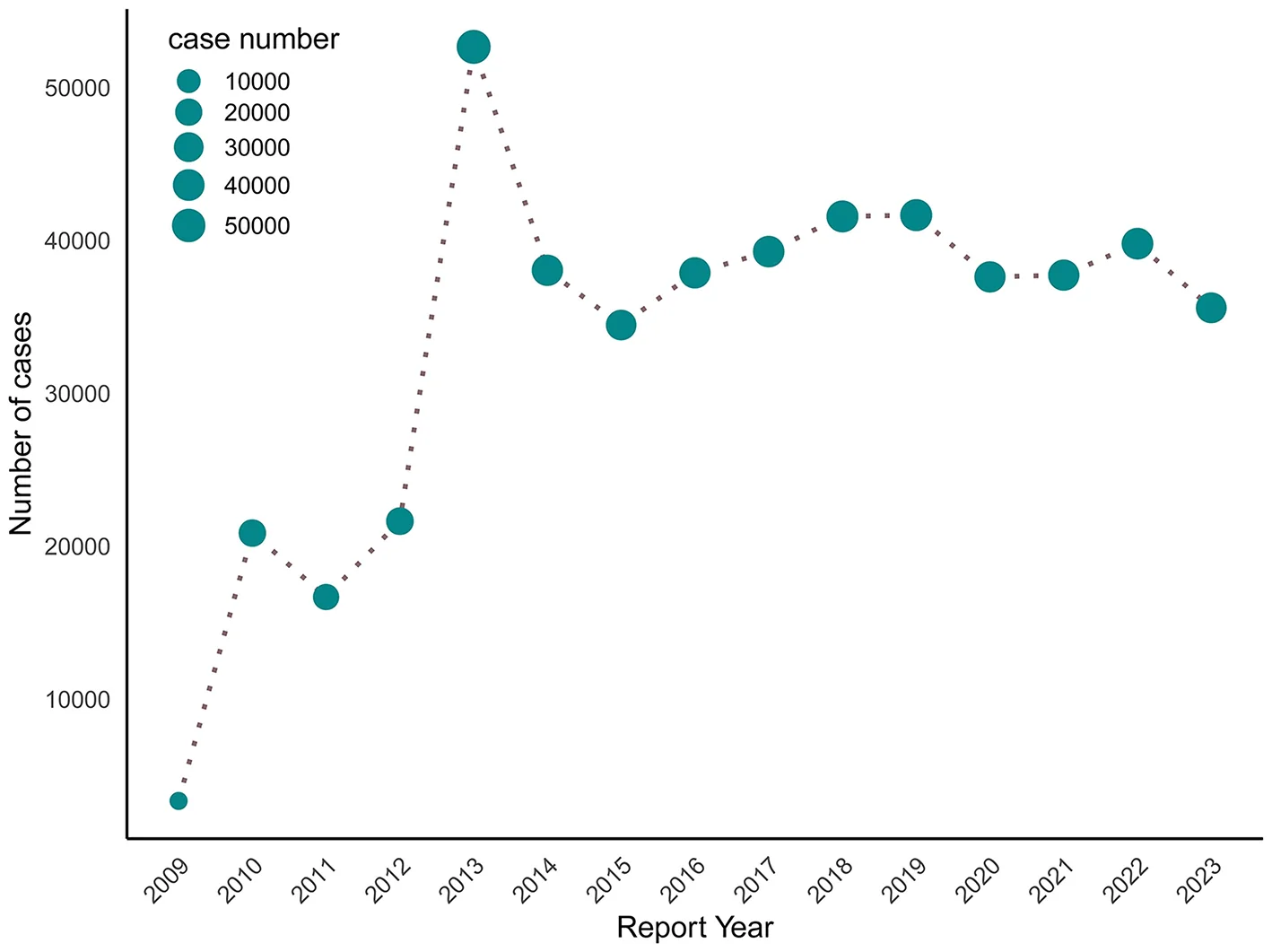
Annual number of adverse drug events (ADEs) reported from the first quarter of 2009 to the fourth quarter of 2023.

Continuous variables were summarized using the mean and standard deviation or the median and interquartile range, depending on the distribution of the data. Categorical variables were summarized using frequencies and percentages. The proportion of missing values was reported for variables with substantial missingness. No statistical imputation was performed for missing demographic or report-level information.

Descriptive analyses were intended to characterize the composition of the reporting dataset and were not interpreted as estimates of the demographic distribution of the underlying MS population or of the treated population. Differences in the number of reports across drugs may reflect differences in market availability, prescribing frequency, treatment duration, patient characteristics, and reporting behavior.

### 2.3 Disproportionality analysis of ADEs

To identify ADEs associated with the eight DMT categories, we applied two frequentist algorithms: reporting odds ratio (ROR) (18) and proportional reporting ratio (PRR) (19), as well as two Bayesian algorithms: Bayesian Confidence Propagation Neural Network (BCPNN) (20) and multi-item gamma Poisson shrinker (MGPS) (21). For each drug–event combination, a 2 × 2 contingency table was constructed. The table cross-classified reports according to whether they involved the drug of interest and the event of interest, compared with reports involving other selected drugs and/or other reported events. The exact definition of the four cells and the corresponding calculation formulas are provided in Supplementary Table S2.

Each of these algorithms has its advantages: frequentist methods are more effective in detecting early signals of ADEs (22–24), while Frequentist and Bayesian approaches have different statistical properties and may provide complementary information for signal detection. Bayesian shrinkage methods can reduce the influence of random variation, particularly for drug–event combinations with small observed counts (25). The use of multiple algorithms was intended to evaluate the robustness and reproducibility of potential signals rather than to generate independent estimates of clinical risk.

All algorithms were based on 2 × 2 contingency tables, and the detailed calculation methods and thresholds are provided in Supplementary Table S2. A drug–event combination was considered a potential signal in the primary analysis only when it met the predefined threshold for all four algorithms. The rationale for these thresholds and the corresponding references are provided in Supplementary Table S2.

ADE signals that exceeded the threshold for each algorithm were considered indicative of a potential association between the drug and the ADE. After conducting disproportionality analyses at both the SOC and PT levels, we selected ADE signals that met the threshold for all four algorithms to ensure reliability. The strength of the signal determined the degree of association between the drug and the ADE.

The magnitude of an ROR, PRR, information component, or empirical Bayes estimate reflects the degree of disproportionate reporting within FAERS. It does not represent an incidence rate, relative risk, hazard ratio, or attributable risk. A signal was therefore interpreted as a screening-level association requiring further evaluation, rather than as evidence of a causal relationship. For drug-specific comparisons, the analyses were conducted within the same FAERS reporting framework and should be interpreted cautiously. Differences in reporting disproportionality may be affected by differences in prescribing volume, time since marketing authorization, patient population, disease severity, treatment duration, concomitant medications, and reporting intensity.

No multiplicity-adjusted causal inference was intended. The four-algorithm intersection was used as a conservative signal-stability criterion; it does not eliminate residual reporting bias or establish causality.

### 2.4 Subgroup analysis

The FAERS database provides demographic data on sex, age, and weight. To explore differences in ADEs among different populations using DMT, we performed subgroup analyses based on these demographic variables. The subgroup analysis used the same four disproportionality algorithms and was conducted separately for sex (male, female), age (< 60 years, ≥60 years), and weight (< 70 kg, ≥70 kg). The subgroup cutoffs were selected according to the distribution of available data and their clinical interpretability. Because the subgroup analyses were based on reported rather than population-based exposure data, they were considered exploratory. For each subgroup analysis, reports with missing information for the subgroup-defining variable were excluded from that specific subgroup comparison but were not imputed. The same drug and event definitions, PS criteria, deduplication procedures, and signal thresholds used in the primary analysis were applied to the subgroup analyses. Volcano and forest plots were generated to compare ADE differences between different population groups, based on ROR and *p*-values. The graphical comparisons were used to display differences in reporting disproportionality and were not interpreted as direct comparisons of absolute clinical risk.

Additionally, we conducted disproportionality analyses for each of the eight DMT categories to compare the characteristics of ADEs across different drug types. Based on the frequency and occurrence of various ADEs, we highlighted some characteristic ADEs for each drug type and presented the results using bubble charts.

### 2.5 Time-to-onset probability density function

To characterize the distribution of time from treatment initiation to the reported occurrence of an ADE, we calculated the time interval between the drug therapy start date and the event/report date. Time-to-onset (TTO) was calculated in days using the available THER and DEMO/CASE report-date information. Only records with valid and chronologically plausible dates were included. Reports with missing, inconsistent, or implausible treatment-start or event/report dates were excluded from the TTO analysis.

After excluding cases with inaccurate TTO records, we used the Weibull distribution to calculate the probability density function for TTO. The Weibull distribution is a continuous probability distribution that can describe how risk increases or decreases over time. The distribution's key parameters are the shape parameter (α) and the scale parameter (β), both of which must be greater than zero. α controls the skewness, while β controls the width of the distribution. The estimated shape parameter was accompanied by a 97.5% confidence interval, consistent with the prespecified analysis framework. The probability density formula is provided in Supplementary Table S3.

In this study, the Weibull distribution was used to describe the TTO probability density function, with a focus on the α value. The α parameter was interpreted as a descriptive indicator of the temporal pattern of reported events, rather than as a direct estimate of changing clinical risk. A larger α indicates a right-skewed failure curve, implying a longer TTO. When α < 1 and the 97.5% confidence interval (CI) is < 1, the ADE follows an early-failure curve, where the risk decreases over time. When α = 1, the risk remains constant over time, indicating a random failure curve. When α > 1 and the 97.5% CI is > 1, the risk increases over time, indicating a wear-out failure curve. If the confidence interval included 1, the temporal pattern was considered statistically indeterminate and was not classified as either an early-failure or wear-out pattern. These classifications describe the distribution of reported time-to-onset values and should not be interpreted as proof that the biological risk increases or decreases over time.

TTO analyses were conducted for IMEs, viral infections, elevated liver enzymes, lymphopenia, bacterial urinary tract infections, hair loss, spontaneous abortion, and PML, highlighting TTO for these ADEs across different drug types. Because treatment start dates are incompletely recorded in FAERS and because reports may be submitted at variable times after the event, TTO estimates may be affected by missing dates, recall error, delayed reporting, left truncation, and selective recording. Accordingly, TTO results were interpreted as exploratory descriptions of reported event timing.

### 2.6 Logistic regression analysis

To explore the association between patient characteristics and IME occurrence, we conducted both univariate and multivariate logistic regression analyses. The unit of analysis was the unique FAERS case report after deduplication. Because FAERS may contain multiple reports from the same patient or follow-up versions of a case, the analyses should not be interpreted as patient-level causal analyses.

Independent variables included sex (male, female), age (< 60 years, ≥60 years), weight (< 70 kg, ≥70 kg), and treatment duration (< 1 year, 1–2 years, ≥2 years), while the dependent variable was IME occurrence. The dependent variable was coded as a binary outcome according to whether the report included at least one event classified as an IME. Odds ratios (ORs) for IME occurrence under different exposure conditions were calculated. The regression models were conducted among reports with complete information for the variables included in the respective model. Missing values were not imputed. The number of reports included in each model was reported. The resulting ORs describe associations between report characteristics and the presence of an IME among submitted FAERS reports. They should not be interpreted as population-level risk estimates, incidence rate ratios, or causal effects. In particular, the analyses do not account for the absence of an unexposed comparison cohort or for the complex reporting mechanisms underlying FAERS.

Additionally, we conducted logistic regression analyses for the occurrence of viral infections, elevated liver enzymes, lymphopenia, bacterial urinary tract infections, hair loss, and PML to further explore the relationship between patient characteristics and ADEs. These additional models were considered exploratory. Because multiple outcomes and predictors were examined, the results were interpreted according to the direction, magnitude, and precision of the associations, together with their clinical plausibility and consistency with existing evidence, rather than on statistical significance alone.

## 3 Results

### 3.1 Descriptive analysis

We analyzed FAERS reports submitted from the first quarter of 2009 through the fourth quarter of 2023. After applying the predefined selection criteria, 498,847 cases involving 1,048,575 ADE records were included. The annual number of reports increased substantially during the study period, rising from fewer than 10,000 cases per year in 2009–2013 to more than 50,000 cases annually at its peak and subsequently remaining at approximately 40,000 cases per year.

Among reports with available demographic information, females and middle-aged individuals represented the majority of cases. Reports were predominantly submitted from North America and Western Europe, whereas reports from Asia accounted for a relatively small proportion. Healthcare professionals submitted approximately one-third of the reports. Nearly half of the DMT-related cases involved IMEs, highlighting the substantial proportion of serious or medically important outcomes in the analyzed reporting population. Detailed demographic characteristics are provided in Table 1.

**Table 1:** Demographic characteristics of all ADE cases.

Characteristics	*N* = 498,847^a^
Age	67 (15)
< 18 years	1,977 (0.7%)
>=60 years	47,458 (17%)
18–60 years	227,022 (82%)
Unknown	222,390
Weight	82 (27)
< 70 KG	18,883 (41%)
>=70 KG	26,888 (59%)
Unknown	453,076
Sex	
Female	366,096 (77%)
Male	106,659 (23%)
Unknown	26,092
Reported person	
Health professional Physician (MD)	86,674 (18%)
Non-healthcare professional Consumer (CN)	322,194 (65%)
Pharmacist (PH)	15,676 (3.2%)
Health professional (HP)	22,018 (4.5%)
Other health professional (OT)	46,790 (9.5%)
Lawyer (LW)	40 (< 0.1%)
Unknown	5,221
Reported country	
US	389,772 (78%)
DE	14,091 (2.8%)
CA	10,923 (2.2%)
UK	5,753 (1.2%)
FR	4,322 (0.9%)
IT	4,231 (0.8%)
AU	3,048 (0.6%)
BR	2,896 (0.6%)
NL	2,064 (0.4%)
JP	1,704 (0.3%)
BE	1,418 (0.3%)
IRL	1,048 (0.2%)
EG	1,017 (0.2%)
Others	56,525 (11.3%)
Unknown	35
Outcome	
Hospitalization-Initial or prolonged (HO)	78,766 (41%)
Other (OT)	93,776 (49%)
Death (DE)	11,064 (5.8%)
Life-threatening (LT)	3,655 (1.9%)
Disability (DS)	2,659 (1.4%)
Required intervention to prevent permanent impairment/damage (RI)	176 (< 0.1%)
Unknown	308,238

^a^Mean (SD); *n* (%).

### 3.2 ADE signal surveillance at the SOC level

At the SOC level, nervous system disorders and general disorders and administration site conditions were the most frequently reported categories, followed by infections and infestations, gastrointestinal disorders, musculoskeletal and connective tissue disorders, and injury-, poisoning-, and procedure-related complications. The most prominent disproportionality signals were observed for vascular disorders and nervous system disorders, while infections and infestations also showed a positive reporting association. General disorders and administration site conditions demonstrated a relatively weak but statistically detectable signal. The principal SOC-level ROR estimates and confidence intervals are presented in Figures 3A, B and Supplementary Table S4.

**Figure 3:**
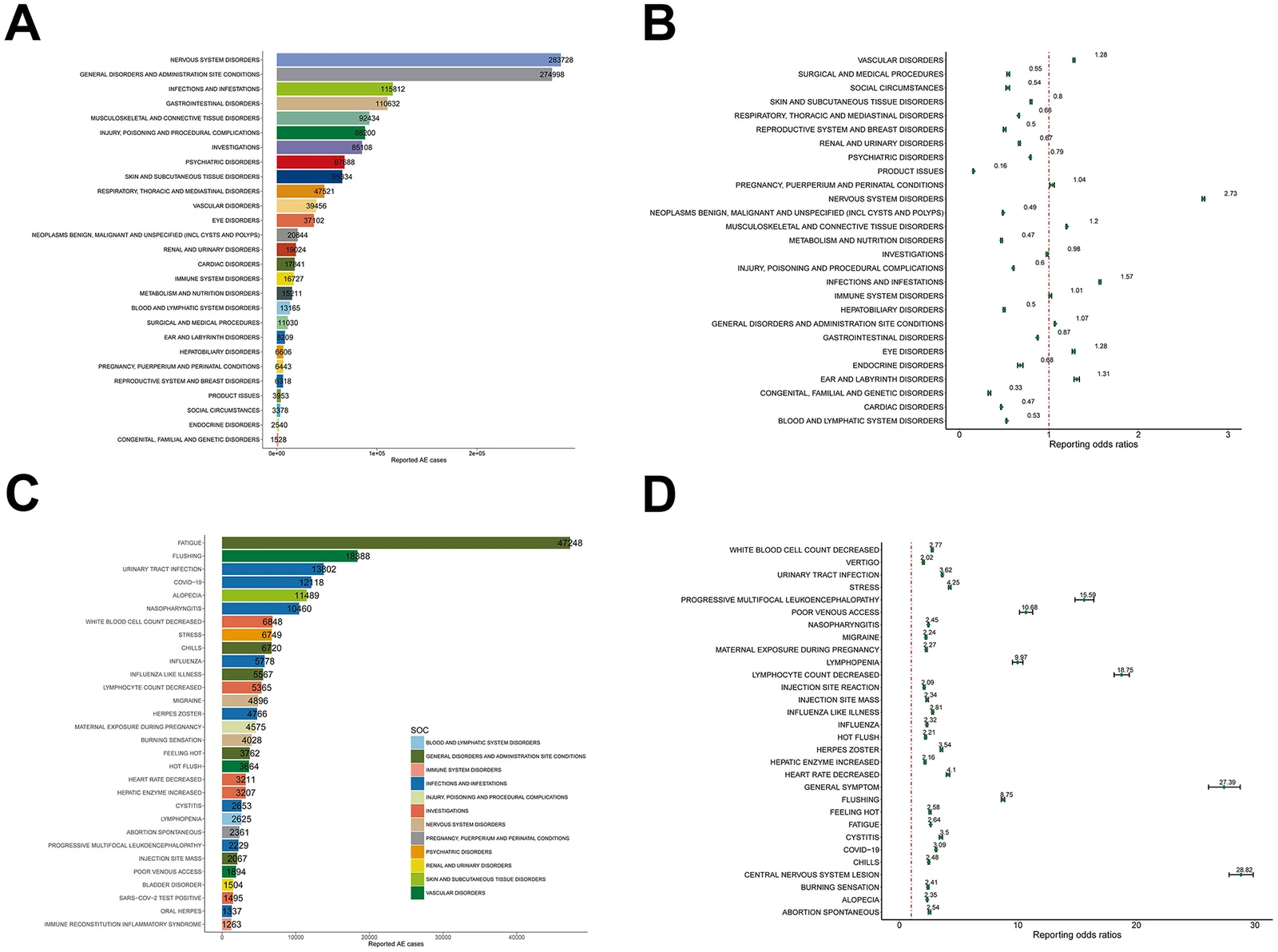
Results of ADE signal monitoring. **(A, B)** ADE signal monitoring results at the System Organ Class (SOC) level, including a frequency distribution histogram **(A)** and a forest plot based on the reporting odds ratio (ROR) **(B)**; **(C, D)** ADE signal monitoring results at the Preferred Term (PT) level, including a frequency distribution histogram **(C)** and a forest plot based on ROR **(D)**.

Overall, the SOC-level analysis suggested that the post-marketing safety profile of MS DMTs was characterized by three broad domains: neurological events, immune- or infection-related events, and treatment-related systemic or administration-site conditions. These findings provide a high-level framework for interpreting the more specific PT-level signals.

### 3.3 ADE signal surveillance at the PT level

At the PT level, after excluding symptoms and etiologies closely related to MS, the most frequently reported events were nonspecific systemic symptoms, including fatigue, headache, nausea, flushing, and asthenia. Hematological abnormalities, particularly decreased white blood cell and lymphocyte counts, were also commonly reported and were consistent with the immunomodulatory effects of several DMTs. Infection-related PTs, including urinary tract infection, COVID-19, nasopharyngitis, influenza, herpes zoster, and general infection, represented another major reporting domain. Liver-related laboratory abnormalities, such as increased hepatic enzymes, alanine aminotransferase, and aspartate aminotransferase, were also observed.

Among the clinically important signals, PML and central nervous system lesions showed the largest disproportionality estimates, followed by decreased lymphocyte count. Other notable signals included urinary tract infection, COVID-19, spontaneous abortion, alopecia, and increased hepatic enzyme levels. The corresponding event counts and disproportionality estimates are provided in Figures 3C, D and Supplementary Table S5.

Taken together, the PT-level findings clustered into four main categories: general systemic symptoms, hematological or hepatic laboratory abnormalities, infections and infestations, and rare but clinically serious neurological or pregnancy-related events. This pattern was broadly compatible with the immune-modulating mechanisms and known safety concerns of MS DMTs, although the FAERS data cannot determine absolute incidence or establish causality.

### 3.4 Subgroup analysis

Subgroup analyses evaluated differences in ADE reporting patterns according to sex, age, body weight, and DMT category. These analyses compared reporting patterns between subgroups and were not designed to estimate incidence or establish causal associations.

Sex-related differences were observed across several event domains. Females had higher reporting of alopecia, migraine, fungal infections, infusion-related reactions, and viral infections, whereas males showed relatively higher reporting of selected hepatic, cardiovascular, and neurological events according to the subgroup estimates. Age-stratified analyses also showed distinct reporting profiles: younger and older patients differed in the reporting of cardiovascular, infectious, hepatic, hematological, and neurological events. Weight-stratified analyses identified differences in the reporting of diabetes mellitus, COVID-19 pneumonia, deep vein thrombosis, lymphocyte-count decreases, neutropenia, breast cancer, and spontaneous abortion. The principal subgroup estimates are shown in Figure 4.

**Figure 4:**
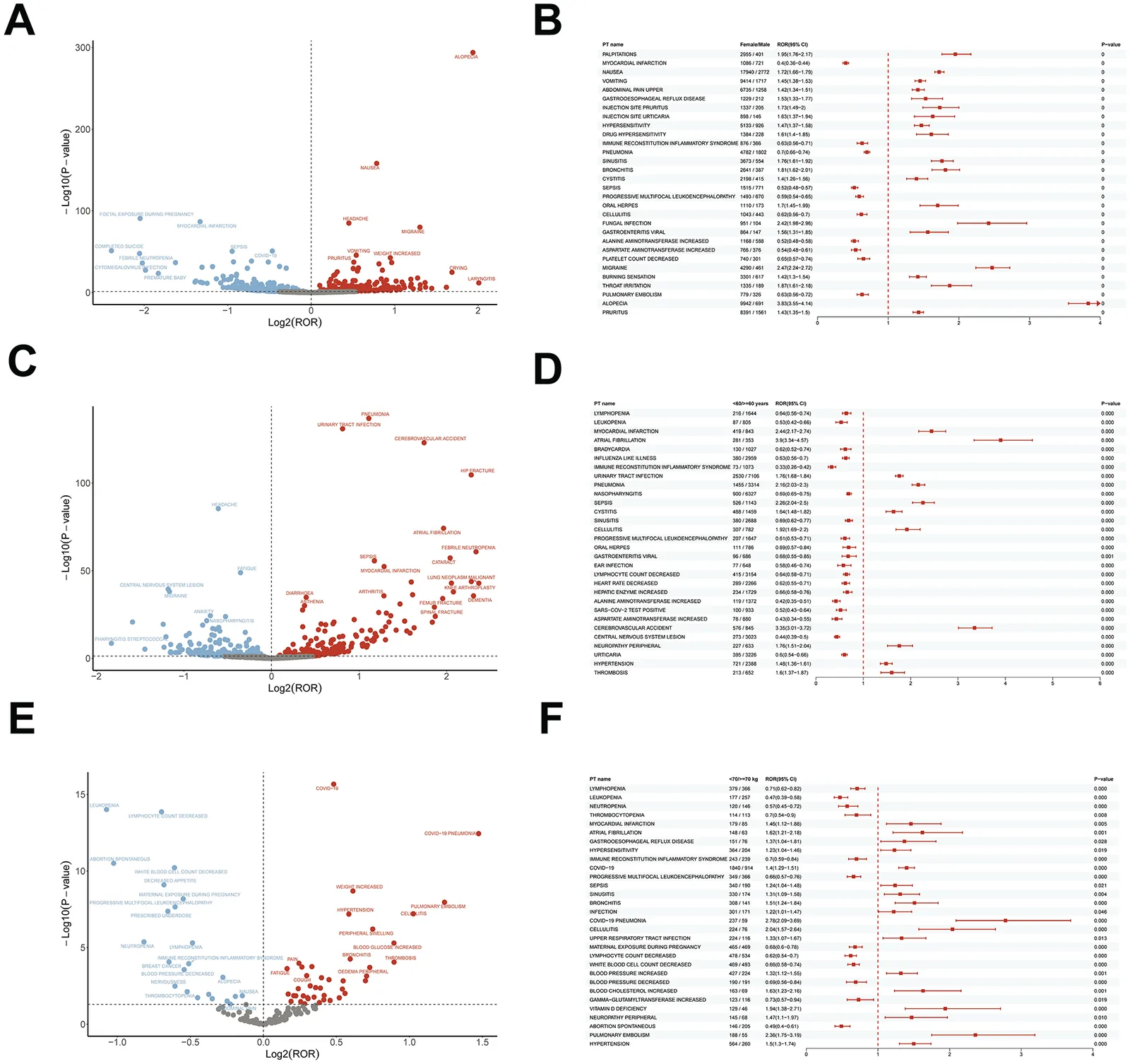
Results of subgroup analysis based on age, gender, and weight. **(A, B)** Subgroup analysis by age based on ROR, including a volcano plot **(A)** and a forest plot **(B)** according to the differential analysis results; **(C, D)** Subgroup analysis by gender based on ROR, including a volcano plot **(C)** and a forest plot **(D)** according to the differential analysis results; **(E, F)** Subgroup analysis by weight based on ROR, including a volcano plot **(E)** and a forest plot **(F)** according to the differential analysis results.

These findings indicate that the distribution of reported ADEs varied across demographic subgroups, but they do not establish demographic differences in absolute clinical risk. The observed patterns may reflect biological susceptibility, treatment selection, disease characteristics, comorbidities, concomitant medications, monitoring intensity, and reporting behavior.

### 3.5 Subgroup analysis by drug type

The reporting profiles differed substantially across the eight DMT categories. Infection-related signals were particularly prominent among anti-CD20 monoclonal antibodies, especially for COVID-19 and bacterial urinary tract infections. Anti-CD52 monoclonal antibodies showed a strong reporting signal for lymphocyte-count decreases, whereas natalizumab was most strongly associated with PML and other central nervous system lesions. S1PR modulators were also characterized by prominent reports of lymphocyte-count decreases and non-PML central nervous system lesions.

Other therapies showed more treatment-specific patterns. Glatiramer acetate was mainly associated with injection-site reactions and injection-site erythema. NRF2 agonists showed a pattern involving cutaneous and gastrointestinal events, including rash, flushing, vomiting, abdominal pain, and diarrhea. Teriflunomide was particularly associated with alopecia. Anti-CD25 monoclonal antibodies showed signals involving infections and liver-related events. Detailed event counts and ROR estimates for each drug category are provided in Figure 5A and Supplementary Table S6.

**Figure 5:**
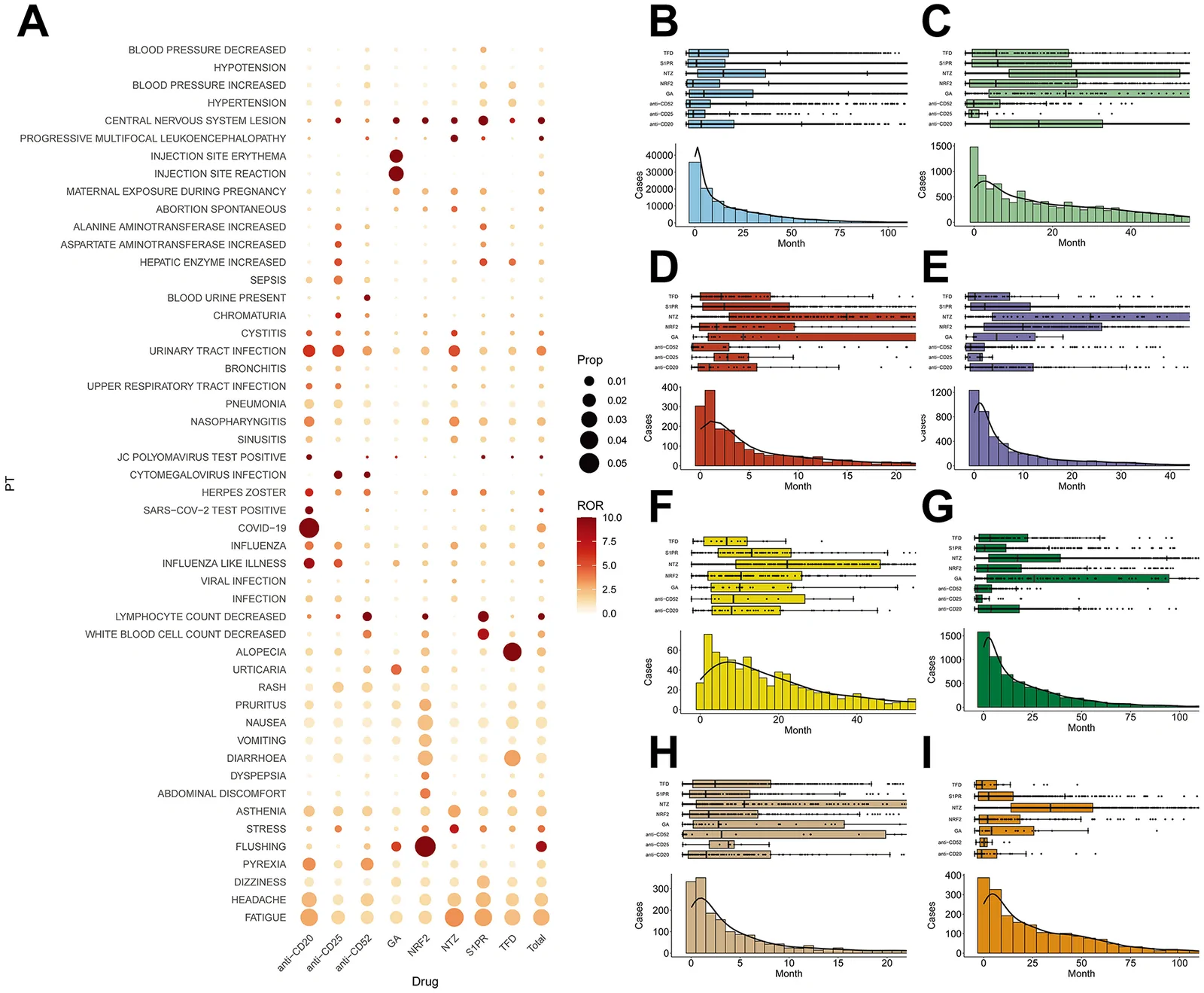
Subgroup analysis and Time-to-Onset (TTO) analysis based on different drug types. **(A)** Characteristics of ADEs for different types of drugs; **(B)** TTO frequency distribution for important medical events; **(C)** TTO frequency distribution for viral infections; **(D)** TTO frequency distribution for elevated liver enzymes; **(E)** TTO frequency distribution for lymphopenia; **(F)** TTO frequency distribution for spontaneous abortion; **(G)** TTO frequency distribution for urinary tract infections; **(H)** TTO frequency distribution for alopecia; **(I)** TTO frequency distribution for progressive multifocal leukoencephalopathy (PML).

Thus, the drug-class analysis suggested mechanism- and treatment-specific reporting profiles rather than a uniform safety pattern across DMTs. These results may help prioritize therapy-specific monitoring but should not be interpreted as a comparative safety ranking or as evidence that one DMT is clinically safer than another.

### 3.6 Time-to-onset analysis

Among 116,952 reports with complete treatment-duration information, approximately 70% of IMEs were reported within two years after treatment initiation. The median TTO for IMEs was 300 days, and the Weibull distribution showed an early-failure pattern, with a decreasing probability of reporting over time (shape parameter α = 0.66; scale parameter β = 462.22). The shortest median TTOs were observed for anti-CD52 monoclonal antibodies, anti-CD25 monoclonal antibodies, and NRF2 agonists, whereas natalizumab had the longest median TTO.

The TTO distributions of most selected ADE domains, including viral infections, elevated liver enzymes, lymphopenia, bacterial urinary tract infections, alopecia, and PML, also generally showed early-failure patterns. Median TTOs were shortest for alopecia, elevated liver enzymes, and lymphopenia, whereas spontaneous abortion showed a less clearly time-dependent pattern. PML had a median TTO of 550 days and was most frequently associated with natalizumab, for which the median TTO was approximately 40 months. The corresponding TTO estimates are summarized in Table 2 and Figures 5B–I.

**Table 2:** Parameters for calculating the probability density function of time-to-onset based on the Weibull distribution.

Group	Number	TTO (days)		Weibull distribution				Failure type
				Shape parameter		Scale parameter		
		Median (IQR)	Min-max	α	97.5% CI	β	97.5% CI	
Total	116,952	300.00 (56.00–867.00)	1–20,455	0.66	0.65–0.66	462.22	457.97–466.47	Early failure
Viral infections	11,857	490.00 (143.00–1,060.00)	1–7,762	0.81	0.79–0.82	648.05	632.89–663.21	Early failure
Elevated liver enzymes	2,089	101.00 (31.00–345.00)	1–15,711	0.63	0.61–0.65	223.34	207.33–239.36	Early failure
Lymphopenia	4,762	117.00 (30.00–393.00)	1–7,196	0.64	0.62–0.65	233.91	222.89–244.93	Early failure
Spontaneous abortion	857	505.00 (211.00–1,046.00)	1–6,711	0.99	0.94–1.04	751.35	697.77–804.93	Random failure
Urinary tract infections	6,065	365.00 (84.00–933.00)	1–14,517	0.71	0.70–0.72	546.42	526.06–566.78	Early failure
Alopecia	1890	90.00 (25.00–271.00)	1–5,478	0.63	0.61–0.65	184.15	170.26–198.05	Early failure
PML	1985	550.00 (139.00–1,312.00)	1–6,271	0.81	0.78–084	755.58	712.46–798.69	Early failure

Overall, the TTO analysis suggested that many DMT-associated IMEs and ADEs were reported relatively early after treatment initiation, although some clinically important events, particularly PML, may be reported after prolonged exposure. These findings may inform the timing of clinical vigilance but cannot be interpreted as direct estimates of event latency or causal hazard because treatment initiation dates and event dates in spontaneous reports may be incomplete, imprecise, or affected by reporting delay.

### 3.7 Logistic regression analysis

In the analysis of overall IME reporting, older age, male sex, and longer treatment duration were associated with higher odds of IME reports, whereas higher body weight was associated with lower reporting odds. In event-specific analyses, male sex was associated with higher reporting of elevated hepatic enzymes but lower reporting of bacterial urinary tract infection and alopecia. Older age was associated with higher reporting of bacterial urinary tract infection but lower reporting of elevated hepatic enzymes, lymphopenia, and PML. Higher body weight was associated with lower reporting of lymphopenia and PML.

Treatment duration showed event-specific associations. Compared with treatment duration of less than one year, treatment exceeding one year was associated with higher reporting of viral infections and PML, whereas longer treatment duration was associated with lower reporting of elevated hepatic enzymes, lymphopenia, and alopecia. Treatment duration of one to two years was associated with higher reporting of bacterial urinary tract infection than treatment duration of less than one year or more than two years. The full univariate and multivariate estimates are presented in Figures 6A–N.

**Figure 6:**
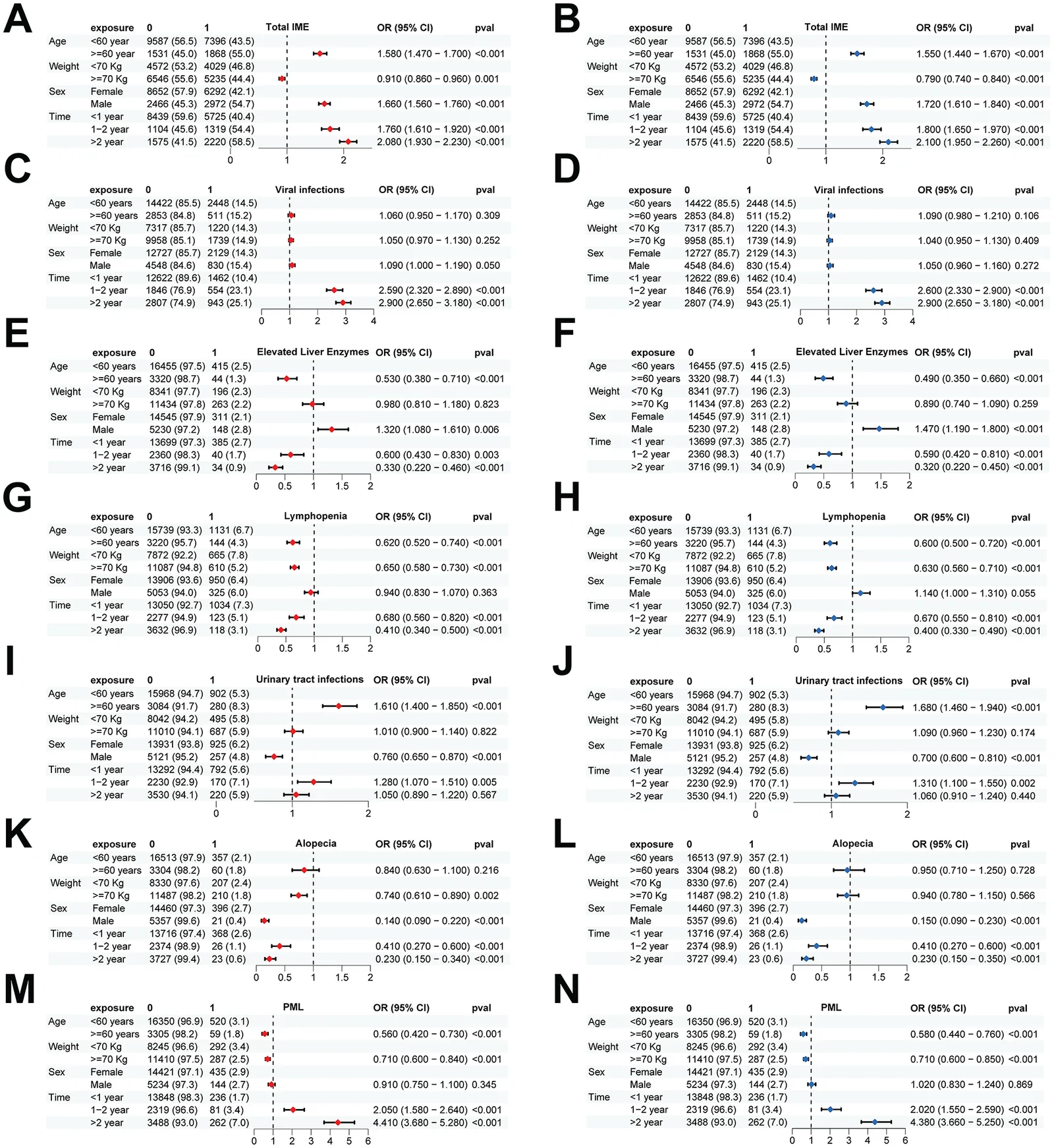
Results of univariate and multivariate logistic regression based on age, gender, weight, and duration of medication. **(A, B)** Forest plot of univariate **(A)** and multivariate **(B)** logistic regression with IMEs as the dependent variable; **(C, D)** Forest plot of univariate **(C)** and multivariate **(D)** logistic regression with viral infections as the dependent variable; **(E, F)** Forest plot of univariate **(E)** and multivariate **(F)** logistic regression with elevated liver enzymes as the dependent variable; **(G, H)** Forest plot of univariate **(G)** and multivariate **(H)** logistic regression with lymphopenia as the dependent variable; **(I, J)** Forest plot of univariate **(I)** and multivariate **(J)** logistic regression with urinary tract infections as the dependent variable; **(K, L)** Forest plot of univariate **(K)** and multivariate **(L)** logistic regression with alopecia as the dependent variable; **(M, N)** Forest plot of univariate **(M)** and multivariate **(N)** logistic regression with PML as the dependent variable.

These findings indicate that the factors associated with IME reporting were not uniform across event types. However, because the analyses were conducted using spontaneous reports without a non-exposed comparison group and with incomplete adjustment for patient-level confounders, the regression results should be considered exploratory associations rather than causal risk estimates or individual-level prediction models.

## 4 Discussion

MS is a chronic autoimmune disease that primarily affects the central nervous system (CNS). It is characterized by inflammatory damage to the protective coverings of nerves in the brain and spinal cord, leading to slowed or completely disrupted nerve signal transmission. This results in a range of neurological symptoms, a decline in quality of life, and poor outcomes such as disability. Currently, there is no cure for MS. DMT aims to reduce the risk of relapse and potential disability progression by modulating the immune system. However, the complex mechanisms of action of immunomodulators, the heterogeneity of the disease, the need for regular monitoring during treatment, and the occurrence of ADEs pose significant challenges to clinical decision-making. This makes achieving ideal personalized treatment difficult. Patients and clinicians must reach a consensus on treatment plans by balancing efficacy, side effects, and potential risks (26).

The immune system plays a critical role in maintaining homeostasis and eliminating pathogens. In the pathogenesis of MS, peripheral immune responses drive the early stages of the disease, while CNS immune responses dominate its progression. T cells, which are key players in adaptive immunity, are widely recognized as central to MS pathology. Additionally, the crosstalk between inflammatory responses and metabolic pathways adds another layer of complexity to treatment (27). DMT refers to therapies used during the remission phase of MS and includes a range of immunomodulators, from broad-spectrum immune regulators to targeted therapies aimed at specific immune pathways. For example, fingolimod reduces inflammatory disease activity by inhibiting lymphocyte migration (28), while NTZ mitigates CNS inflammation by decreasing blood-brain barrier permeability (29). Monoclonal antibodies targeting CD20 and CD52 deplete lymphocytes to limit inflammatory progression (30–32). However, manipulating immune balance with these therapies can introduce new disease risks, leading to an increase in ADEs. Real-world investigations into the ADE profiles of DMTs are crucial for improving the comprehensive management of MS.

In this study, we conducted a 15-year real-world pharmacovigilance analysis based on the FAERS database, focusing on DMT-associated ADEs. The results revealed that the most frequently reported PTs for ADEs were various somatic symptoms, reductions in immune cell counts, and declines in liver function markers. These findings are broadly compatible with the known pharmacological effects and monitoring requirements of several DMTs; however, the frequency of reporting and the strength of disproportionality signals should not be interpreted as measures of incidence or absolute clinical risk. Notably, we identified several severe ADEs, including infections and infestations, spontaneous abortion, and CNS damage, particularly PML.

The high ROR values observed for some drug–event combinations should be interpreted as measures of disproportionate reporting rather than direct estimates of clinical risk. Particularly for rare events, a relatively small number of reports in the exposed cell can produce a large ROR, and the magnitude of the estimate may be influenced by the width of its confidence interval, the size of the background reporting population, and the completeness of case ascertainment. Therefore, the clinical importance of a signal should be assessed jointly according to the number of reports, the precision of the estimate, consistency across ROR, PRR, BCPNN, and MGPS, the seriousness and preventability of the event, and its biological plausibility. Several of the major signals identified in this study are biologically plausible and consistent with the known pharmacology of MS DMTs. The increased reporting of infections, lymphopenia, and other reductions in immune-cell counts is compatible with the immunomodulatory or lymphocyte-depleting effects of therapies targeting lymphocyte trafficking, CD20, CD25, or CD52. Reports involving elevated liver enzymes are also clinically plausible for DMTs that require hepatic monitoring, whereas alopecia has been described in association with selected immunomodulatory therapies. The PML-related signals are consistent with the known risk of impaired immune surveillance and JC virus reactivation during treatment with certain immunosuppressive or lymphocyte-targeted agents, particularly natalizumab and selected lymphocyte-depleting therapies (11). These concordances with established pharmacological mechanisms and previous clinical or post-marketing observations support the value of the findings as safety signals. Nevertheless, biological plausibility does not establish causality. The observed signals may also be influenced by disease severity, treatment selection, concomitant immunosuppressants, comorbidities, differential monitoring, stimulated reporting, and reporting delay. In addition, a high ROR does not necessarily indicate a high absolute risk, especially when the event is rare or when the exposure denominator is unknown. For pregnancy-related outcomes such as spontaneous abortion, the association should be interpreted even more cautiously because pregnancy intention, gestational age, maternal comorbidities, disease activity, concomitant medications, and outcome confirmation may be incompletely recorded in FAERS. Compared with previous pharmacovigilance and clinical studies, the present findings are broadly consistent with the previously recognized safety concerns of infection, hematological abnormalities, hepatic events, and PML associated with immune-modifying therapies (8, 12). The present analysis extends this evidence by evaluating multiple DMT categories within the same FAERS framework and by examining the consistency of signals across multiple algorithms, demographic subgroups, and time-to-onset patterns. However, the findings should be regarded as supportive and hypothesis-generating rather than as evidence of a new causal association or a definitive safety ranking among DMTs. Accordingly, the clinical interpretation of a high ROR should follow a signal-triage framework: signals involving serious or preventable outcomes, consistent findings across analytical methods, plausible latency patterns, and concordance with previous evidence may deserve priority for clinical vigilance and confirmatory investigation. In contrast, isolated signals based on few reports or with imprecise estimates should be interpreted cautiously and should not independently prompt treatment withdrawal or changes in clinical management.

Epidemiological studies have consistently shown that MS is more frequently diagnosed in women than in men, and our descriptive and subgroup analyses also identified sex-related differences in the reporting patterns of DMT-associated adverse events. Women receiving DMTs showed higher reporting of fungal and viral infections and alopecia, while logistic regression analysis indicated that, among submitted FAERS reports, women had higher odds of reports involving bacterial urinary tract infections classified as IMEs than men. These findings may be related to sex-related differences in immune regulation, infection susceptibility, healthcare utilization, and treatment monitoring (33, 34). Women generally exhibit stronger innate and adaptive immune responses, which may involve differences in antigen presentation, lymphocyte activation, cytokine production, and sex-hormone-mediated immune modulation (35–37). These biological differences could influence both the clinical occurrence and the likelihood of recognizing and reporting infection-related events. However, the present FAERS analysis cannot determine whether women have a higher absolute risk of these events, because FAERS lacks reliable exposure denominators and is affected by reporting, selection, and confounding biases. Therefore, the sex-specific findings should be interpreted as differences in reporting patterns rather than definitive evidence of sex-specific causal effects. Age-stratified analyses also suggested differences in the reporting patterns of DMT-associated adverse events between younger and older patients. Several clinical and biological factors may contribute to these differences. Older patients may have greater comorbidity burden, more frequent polypharmacy, age-related immune senescence, reduced physiological reserve, and a higher likelihood of receiving laboratory and clinical monitoring. These factors may influence susceptibility to infection, hematological abnormalities, hepatic events, and other serious outcomes, as well as the probability that an event is detected and reported. In contrast, younger patients may differ in treatment selection, disease duration, reproductive considerations, and exposure to high-efficacy DMTs. Age may also be correlated with treatment duration and disease severity, making it difficult to separate the independent effect of age from other clinical characteristics. Accordingly, the age-related findings in this study should be considered exploratory and hypothesis-generating rather than evidence of a causal age-dependent risk. The observed differences by sex and age may therefore reflect a combination of biological susceptibility, treatment selection, disease characteristics, comorbidities, healthcare utilization, and reporting behavior. These factors should be considered when clinicians individualize safety monitoring, but the present results should not be used to rank DMT safety or to recommend treatment modification solely on the basis of sex or age. Previous studies have demonstrated a strong association between infections and spontaneous abortion, involving pathogens such as viruses, mycoplasma, bacteria, and parasites (38–42). While antibiotics may help control infections, some studies suggest that their use during pregnancy does not significantly affect the incidence of spontaneous abortion. However, this lack of association is complicated by factors such as antibiotic type and dosage, timing of conception, and overall patient health (43). The observed reporting signal involving spontaneous abortion should be interpreted cautiously, particularly because pregnancy-related information, disease activity, concomitant treatment, and clinical outcomes may be incompletely recorded in FAERS. It may reflect the underlying effects of infection, MS disease activity, pregnancy-related factors, concomitant medications, selective reporting, or outcome misclassification rather than a direct effect of a specific DMT. Therefore, spontaneous-abortion reports may warrant further evaluation in women of childbearing age receiving DMTs, but the present findings cannot establish a causal relationship or estimate the absolute risk of pregnancy loss.

PML is an opportunistic demyelinating disease of the CNS associated with JC virus infection and reactivation. Previously, it was primarily observed in HIV-infected individuals (44). After initial infection, JC virus persists in a latent state in various tissues, including the tonsils, lymph nodes, bone marrow, spleen, brain, kidneys, and lungs (45–47). With the increasing use of monoclonal antibodies for various diseases, PML cases have also risen in non-HIV populations (48). The underlying mechanism is thought to involve immunosuppressive effects and lymphocyte depletion caused by immunomodulators, leading to JC virus reactivation. Two key findings in this study support this hypothesis: JC virus test positivity was significant in cases involving multiple drugs; and the median TTO for PML was very close to the median TTO for infections. In clinical and post-marketing studies, PML has been reported in approximately 1 in 1,000 patients receiving natalizumab, although the absolute risk varies according to anti-JC virus antibody status, prior immunosuppressant exposure, treatment duration, and other patient-level factors (49). The proposed mechanism involves impaired immune surveillance and reduced trafficking of JC virus-specific lymphocytes, which may facilitate viral reactivation in susceptible patients (50, 51). A similar mechanism is observed with alemtuzumab, a humanized monoclonal antibody targeting the CD52 receptor. Alemtuzumab-associated PML may be linked to cytotoxic T-cell depletion (52, 53), which is unaffected by current prophylactic measures (54). Ofatumumab, which targets the CD20 epitope and primarily acts on B cells, has also been associated with PML, although reports remain scarce (55). In our logistic regression analysis, treatment duration exceeding one year was associated with higher odds of reports involving PML-related IMEs. Because this analysis was conducted within the FAERS reporting population and lacked a non-exposed comparison group, the finding should not be interpreted as evidence that treatment duration causally increases the clinical risk of PML. It may also reflect cumulative exposure, treatment selection, differential monitoring, reporting delay, or confounding by disease severity. The conflict between the necessity of long-term treatment and the associated risks highlights the challenges of managing chronic diseases like MS. Overall, the rapid progression and poor prognosis of PML make it a critical IME requiring careful clinical vigilance during DMT. However, FAERS-based findings cannot determine the comparative risk of PML across DMTs or justify treatment avoidance in individual patients. Clinical decisions should be based on validated risk factors, current treatment guidelines, and individualized benefit–risk assessment.

### 4.1 Novelty and positioning of the present study

The present study should be interpreted in the context of previous FAERS-based pharmacovigilance investigations of MS disease-modifying therapies (DMTs). Although previous studies have provided valuable information on selected drugs, specific adverse-event categories, or particular clinical concerns, many have focused on individual therapies, limited therapeutic classes, or relatively short observation periods. In addition, differences in drug selection, event definitions, analytical methods, and reporting periods make it difficult to obtain a systematic overview of the post-marketing safety-reporting profiles of major MS DMTs within a common analytical framework. The added value of the present study does not rest solely on the size of the database or the number of reports. Rather, its contribution lies in the integration of several complementary dimensions of pharmacovigilance assessment. First, the use of more than 15 years of FAERS data provides an extended observation window that encompasses different stages of clinical use, market exposure, and post-marketing experience for multiple MS DMTs. This longitudinal perspective allows us to characterize the distribution and persistence of reported safety signals across an extended period and to identify safety domains that may warrant continued monitoring. However, the extended observation period does not eliminate temporal changes in drug utilization, treatment indications, patient characteristics, regulatory warnings, or reporting behavior. Second, the study evaluates multiple DMT categories within a common analytical framework. The drugs were grouped according to their primary mechanisms of action, allowing the observed adverse-event reporting patterns to be discussed in relation to broad pharmacological and immunological characteristics. This approach facilitates structured comparisons across therapeutic classes while avoiding the assumption that drugs sharing a primary mechanism have identical safety profiles. The mechanism-based classification is therefore used as an interpretative framework rather than as evidence of a class-wide causal effect. Third, we used both frequentist and Bayesian disproportionality methods and required consistency across four algorithms for the primary signal analysis. This approach was intended to reduce reliance on a single statistical metric and to distinguish more reproducible reporting signals from isolated numerical findings. The use of multiple algorithms and the four-method intersection represent an internal signal-stability strategy within the FAERS dataset. They do not constitute external validation, do not correct for all sources of reporting bias, and should not be confused with confirmation of causality or estimation of comparative clinical risk. Fourth, the present study extends beyond a conventional cross-sectional signal-detection analysis by incorporating demographic subgroup analyses, time-to-onset characterization, and exploratory regression analyses of IMEs and selected adverse-event domains. These additional analyses provide contextual information regarding the distribution of reported events across demographic groups, the timing of reported events after treatment initiation, and the characteristics of reports involving serious outcomes. They were conducted to support clinical interpretation and hypothesis generation, rather than to establish effect modification, temporal causality, or patient-level risk prediction.

Accordingly, the primary contribution of this study is the integration of a long-term reporting window, a broad mechanism-based comparison of major MS DMTs, complementary signal-detection algorithms, and exploratory clinical-context analyses in a single pharmacovigilance framework. To our knowledge, this combined approach provides a broader and more systematic post-marketing safety map than analyses limited to a single DMT, a single adverse-event domain, or one statistical method. Nevertheless, the novelty of the study should be understood as methodological and integrative rather than as the discovery of a definitive causal relationship or the establishment of a safety ranking.

Taken together, the study provides a broad post-marketing safety map of major MS DMTs rather than a definitive ranking of their clinical safety. Its main purpose is to identify consistent, potentially clinically relevant, and prioritizable signals that may guide safety monitoring and inform the design of future investigations. The findings are complementary to randomized trials, cohort studies, pregnancy registries, disease registries, and other longitudinal real-world studies, which are better suited to estimating incidence, comparing absolute risks, evaluating comparative effectiveness, and assessing causality. Therefore, the results should be interpreted as hypothesis-generating evidence that can complement, but not replace, established clinical and epidemiological evidence.

### 4.2 Limitations of the FAERS database and disproportionality analysis

Several limitations inherent to the FAERS database and spontaneous-reporting systems should be considered when interpreting the findings of this study. First, FAERS is subject to underreporting, selective reporting, stimulated reporting, and differences in reporting practices across countries, healthcare systems, reporter types, drugs, and calendar periods. The number of submitted reports therefore cannot be interpreted as the number of exposed patients or as a direct measure of the frequency of an adverse event. In addition, FAERS does not provide a reliable denominator for drug exposure, and the disproportionality measures used in this study cannot be converted into incidence rates, absolute risks, or population-level relative risks (56). Second, although duplicate and follow-up reports were processed according to FAERS-recommended procedures, duplicate submissions and multiple reports referring to the same clinical case may not be completely eliminated. The clinical completeness and accuracy of individual reports also vary considerably. Information on disease duration, MS phenotype, disease activity, treatment indication, treatment adherence, dosage, treatment interruption, concomitant medications, comorbidities, laboratory findings, and clinical follow-up is frequently incomplete or unavailable. These limitations may affect both the identification and the interpretation of drug–event reporting patterns. Third, the observed associations may be affected by confounding by indication and disease severity. Patients receiving high-efficacy or more immunologically targeted DMTs may have more active, severe, or treatment-refractory disease and may undergo more intensive clinical monitoring than patients receiving other therapies. Consequently, some adverse events may reflect the underlying disease, baseline patient characteristics, treatment selection, or differences in healthcare utilization rather than the pharmacological effect of a specific DMT. Concomitant medications and comorbid conditions may introduce additional confounding. These sources of bias cannot be fully controlled using the information available in FAERS. Fourth, the PS classification in FAERS reflects the reporter's assessment that a drug may be suspected of contributing to an event; it does not establish a causal relationship. Similarly, a statistically significant ROR, PRR, BCPNN, or MGPS estimate indicates disproportionate reporting within the database but does not prove causality, biological mechanisms, clinical severity, or increased incidence. The use of four complementary algorithms and the requirement that a signal meet the criteria of all four methods may improve signal consistency, but these procedures cannot eliminate reporting bias or residual confounding (57–59). Therefore, the signals identified in this study should be regarded as hypothesis-generating findings that require confirmation in prospective cohorts, disease registries, comparative epidemiological studies, and other longitudinal real-world data sources. The pregnancy-related and rare neurological findings require particularly cautious interpretation. For example, the reported signal involving abortion or spontaneous abortion cannot be interpreted as evidence that a DMT increases the causal risk of pregnancy loss, because the clinical context, pregnancy intention, disease activity, concomitant treatment, and outcome classification may be incomplete or misclassified in FAERS. Likewise, the identification of PML-related reporting signals indicates a need for continued clinical vigilance but does not provide a direct estimate of PML incidence or comparative risk across DMTs. In this context, the present study is best viewed as a large-scale post-marketing signal-detection analysis that complements, rather than replaces, clinical trials, pregnancy registries, cohort studies, and current treatment guidelines.

### 4.3 Clinical implications and actionable recommendations

Although FAERS signals cannot estimate absolute risk or establish causality, they may help clinicians prioritize safety monitoring during DMT treatment. Before initiating therapy, clinicians should assess disease activity, previous immunosuppressive exposure, comorbidities, concomitant medications, infection history, vaccination status, reproductive plans, and relevant laboratory or cardiovascular risk factors. DMT selection should be based on an individualized benefit–risk assessment and shared decision-making.

During treatment, monitoring should be tailored to the mechanism and known safety profile of the selected DMT. Depending on the specific therapy, this may include periodic blood counts, liver-function tests, cardiovascular assessment, infection surveillance, thyroid monitoring, and evaluation of JC virus-related risk factors. Patients should be informed to promptly report persistent fever, respiratory or urinary symptoms, new neurological symptoms, or other signs of infection.

The pregnancy-related findings support the importance of reproductive counseling and individualized pregnancy planning for women of childbearing potential. However, the present FAERS findings do not establish a causal relationship between any specific DMT and pregnancy loss. Treatment interruption, switching, or discontinuation should therefore be based on clinical evaluation, product-specific evidence, current guidelines, and shared benefit–risk assessment rather than on FAERS signals alone. Overall, the findings are intended to guide surveillance and patient education, not to rank DMT safety or replace clinical judgment.

The exploratory subgroup findings also support a tailored monitoring approach. Clinicians may consider age, sex, reproductive status, comorbidities, concomitant medications, baseline laboratory values, and treatment-specific risk factors when planning follow-up. However, subgroup findings from FAERS should not be used alone to determine treatment eligibility, discontinue a DMT, or infer that one demographic group has a higher absolute risk without confirmatory evidence.

## 5 Conclusion

This study provides a long-term, FAERS-based post-marketing assessment of adverse-event reporting patterns associated with major MS DMTs. By combining mechanism-based drug comparisons, four complementary disproportionality algorithms, demographic subgroup analyses, time-to-onset characterization, and exploratory regression analyses, we identified several potentially important safety-signal domains, including hematological abnormalities, infection-related events, pregnancy-related outcomes, and central nervous system events such as PML. These findings represent disproportionate reporting patterns within FAERS and should not be interpreted as estimates of incidence, comparative risk, or causality. The study is intended to generate hypotheses, support clinical vigilance, and prioritize safety concerns for further evaluation. Confirmation in longitudinal cohorts, disease registries, pregnancy registries, and comparative epidemiological studies is required before these signals can inform comparative treatment decisions.

## Data Availability

The original contributions presented in the study are included in the article/Supplementary material, further inquiries can be directed to the corresponding author.
